# Risk Stratification Tool for Ischemic Stroke: A Risk Assessment Model Based on Traditional Risk Factors Combined With White Matter Lesions and Retinal Vascular Caliber

**DOI:** 10.3389/fneur.2021.696986

**Published:** 2021-08-04

**Authors:** Lu Zhao, Bin Jiang, Hongyang Li, Xiufen Yang, Xiaoyue Cheng, Hui Hong, Yanling Wang

**Affiliations:** ^1^Department of Ophthalmology, Beijing Friendship Hospital, Capital Medical University, Beijing, China; ^2^Department of Neurology, Beijing Friendship Hospital, Capital Medical University, Beijing, China; ^3^Department of Radiology, Beijing Friendship Hospital, Capital Medical University, Beijing, China

**Keywords:** white matter lesions, retinal vessel caliber, ischemic stroke, infarction, risk assessment model

## Abstract

**Objective:** This study aims to establish a risk assessment model based on traditional risk factors combined with the Fazekas classification of white matter lesions and retinal vascular caliber for screening the patients at high risk of ischemic stroke.

**Methods:** This study included 296 patients (128 cases of ischemic stroke and 168 cases in the normal control group). The basic data of the patients were collected. Color fundus photography was performed after pupil dilation, and the retinal vascular caliber was measured using semiautomated vascular measurement software (IVAN Software, Sydney, Australia). The severity of white matter lesions (WML) on cranial nuclear magnetic fluid-attenuated inversion recovery images were assessed using the Fazekas scale. Moreover, logistic regression analysis was used to establish different risk assessment models for ischemic stroke. The effects of models were evaluated through the receiver operating characteristic (ROC) curve and the Delong test compared area under the curve.

**Results:** The sensitivity and specificity of models 1 (the traditional risk factor model), 2 (the retinal vascular caliber model), 3 (the WML model), and 4 (the combined the traditional risk factor, WML and central retinal artery equivalent (CRAE) model) were 71 and 55%, 48 and 71%, 49 and 67%, and 68 and 68.5% with areas under the curve of 0.658, 0.586, 0.601, and 0.708, respectively. The area under the receiver operating characteristic curve in models 1, 2, 3, and 4 showed statistically significant differences. Moreover, no statistical significance exists in the pairwise comparison of other models.

**Conclusion:** The risk assessment model of ischemic stroke combined with Fazekas grade of WML and CRAE is superior to the traditional risk factor and the single-index model. This model is helpful for risk stratification of high-risk stroke patients.

## Introduction

Stroke is a major cause of mortality and long-term disability worldwide (1). The Global Burden of Disease Study published the epidemiological data of vascular diseases in 195 countries and regions from 1990 to 2016 and found that China is one of the countries with the highest incidence of stroke worldwide (2) with 2.5 million new stroke cases annually (3). Moreover, stroke was the leading cause of mortality and disability-adjusted life-years at the national level in China in 2017 (4).

White matter lesions (WML) is a tiny brain vascular lesion with a high signal on imaging performance, and the primary pathological basis is non-inflammatory atherosclerotic changes in the tiny blood vessels of the brain (5). With the development of medical imaging technology, the detection rate of white matter lesions (WML) is continuously increasing. Moreover, WML can be seen in healthy middle-aged and elderly people and can also coexist with ischemic stroke (6), Alzheimer's disease (7), and other cerebrovascular diseases. At the same time, WML has many common risk factors with common cerebrovascular diseases (e.g., advanced age, hypertension, diabetes, atherosclerosis, smoking, and so on) (8). Furthermore, studies found that WML is an independent risk factor for stroke and stroke prediction (9–11).

Retinal blood vessels are the only non-invasive blood vessels in the human body that can be directly observed. However, non-invasive observation and quantification of retinal blood vessels are realized with the development of fundus imaging technology. Retinal abnormalities, as a potential marker of cerebrovascular disease, can be used as a tool for stroke risk stratification to improve stroke risk prediction (12, 13).

Hypertension, diabetes, hyperlipidemia, and smoking are known to be associated with stroke and are risk factors for stroke (14). Similarly, previous studies found that WML and retinal vascular caliber are also associated with stroke. In the current study, the traditional risk factor, retinal vascular caliber, WML, and traditional risk factors combined with WML and retinal vascular caliber models were established to compare the effects of different models of ischemic stroke, aiming to explore a more effective stroke risk stratification method.

## Materials and Methods

### Study Subjects and Design

**Figure 1:**
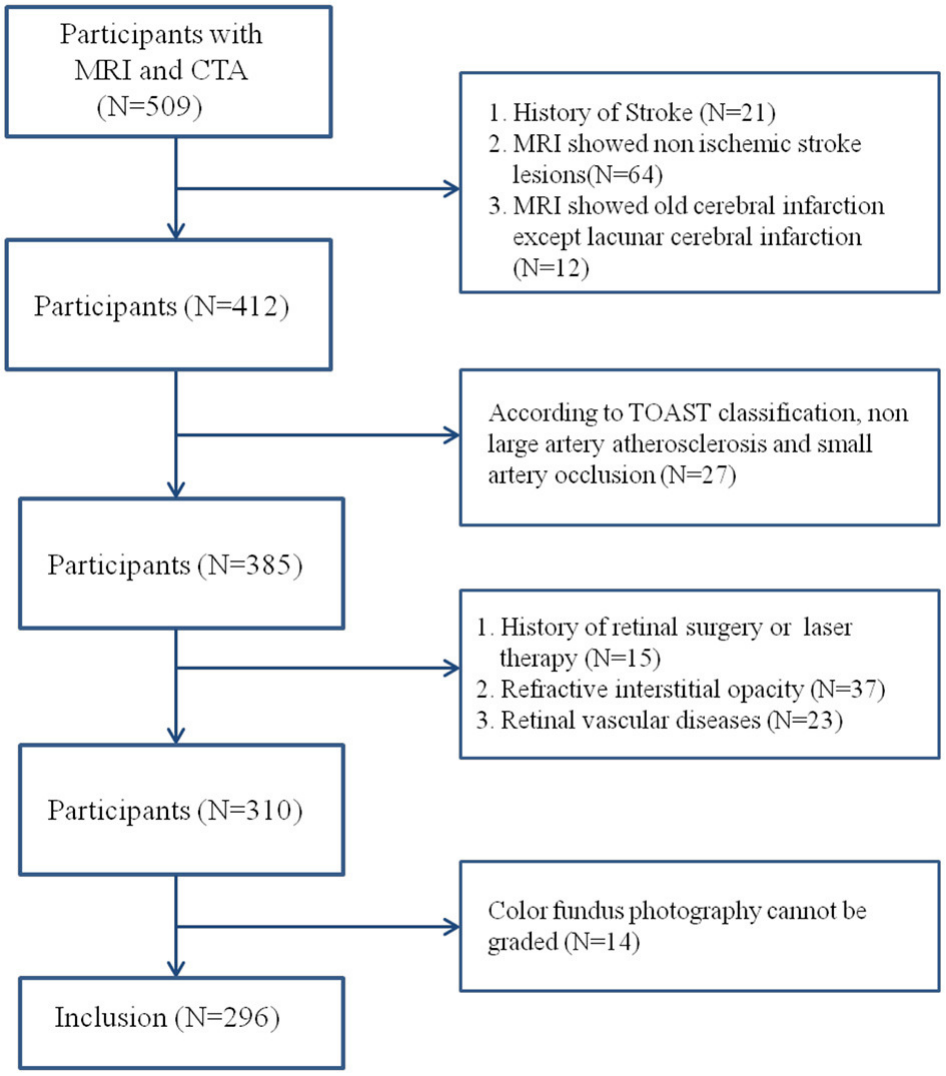
Flowchart of the study.

This was a cross-sectional study that recruited 128 patients with acute ischemic stroke diagnosed in Beijing Friendship Hospital, Capital Medical University, from May 2015 to May 2017. Moreover, 168 non-stroke patients were enrolled as the control group. The inclusion criteria were >50 years old and performed with cranial magnetic resonance imaging (MRI) and head and neck computed tomography angiogram (CTA) examination. The diagnostic classification criteria for ischemic stroke refer to China Guidelines for Diagnosis and Treatment of Acute Ischemic Stroke 2014 and the Trial of Org 10172 in Acute Stroke Treatment (TOAST) and include large-artery atherosclerosis and small-vessel occlusion subtypes (15, 16). The patients in the control group had no history and symptoms of stroke, but had dizziness, headache, transient neurological deficit or disturbance of consciousness. They had brain MRI and head and neck CTA examination, and acute stroke was excluded after neurological examination. MRI and CTA were completed within 1 week after onset, and ophthalmic examination was completed within 1 month after onset. Furthermore, the exclusion criteria were contraindications to MRI or CTA, history of traumatic brain injury and stroke, cerebral hemorrhage and hydrocephalus, and brain tumor or mass. The following ocular diseases were also noted: (1) refractive interstitial opacity which cannot obtain clear fundus images (e.g., severe cataract, keratopathy, and so on), (2) glaucoma, (3) ametropia and diopter >±3.0, (4) uveitis, (5) retinal vascular diseases (e.g., diabetic retinopathy, retinal artery occlusion, and retinal vein occlusion), (6) retinitis pigmentosa, (7) optic neuropathy (e.g., optic neuritis and anterior ischemic optic neuropathy), (8) any history of retinal surgery or laser therapy, and (9) patients who cannot cooperate with the ophthalmic examination (Figure 1).

Demographic data were collected, including age, sex, height, weight, and so on. History related to stroke (history of hypertension, hyperlipidemia, diabetes, smoking, and alcohol consumption), smoking (smoke ≥ 1 cigarette/day on average, continuously or accumulatively for >1 year), and drinking (>50 g/day on average, continuously or cumulatively for >1 year) were also collected. All patients underwent slit-lamp examination and color fundus photography after pupil dilation. The measurement data of the ipsilateral eye were selected from the stroke patients with unilateral lesions, and the data of the right eye or left eye were randomly selected from the stroke patients with bilateral lesions and the control group for statistical analysis using the random number table method.

This study follows the Code of Ethics of The World Medical Association (Declaration of Helsinki) and is approved by the Ethics Committee of Beijing Friendship Hospital, Capital Medical University. Moreover, all patients voluntarily participated and signed the informed consent.

### Cranial MRI Examination and WML Evaluation

The MRI scanner used was GE Signa HDxt 1.5T superconducting magnetic resonance scanner (GE Healthcare, Chicago, IL, USA). All patients completed routine cranial MRI examinations, including T1-weighted imaging, T2-weighted imaging, diffusion-weighted imaging, fluid attenuation inversion recovery, and other sequences.

**Figure 2:**
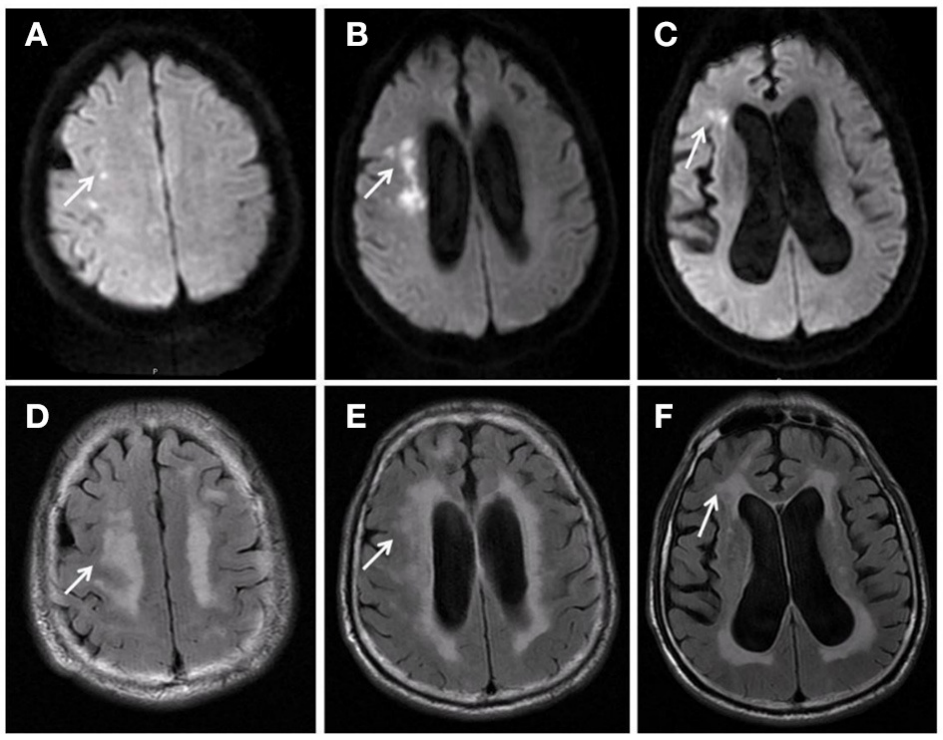
Male, 72 years old. Acute cerebral infarction occurred in the right hemioval center and radial crown **(A–C)**. Fazekas score was 6, paraventricular white matter was 3 **(D)**, and deep-brain white matter was 3 **(E,F)**.

WMH was evaluated blindingly by one experienced neurologist and one experienced radiologist and reassessed by another neurologist when the results were inconsistent. Fazekas scale was used to score WML severity on fluid-attenuated inversion recovery images (17). Paraventricular white matter (0 = no lesion; 1 = cap or pencil-like thin layer lesions; 2 = a halo of smooth lesions; and 3 = paraventricular irregular high signal and extending to deep white matter) and deep-brain white matter (0 = no lesions; 1 = punctate lesion; 2 = lesions start to fuse; 3 = large area fusion of lesions) were evaluated. The sum of the two scores was the total score of Fazekas. Furthermore, Fazekas grade was made according to the sum of the subcortical and bilateral paraventricular lesions: 0, grade 0; 1–2, grade I; 3–4, grade II; and 5–6, grade III (Figure 2).

### Color Fundus Photography and Retinal Vascular Caliber Measurement

**Figure 3:**
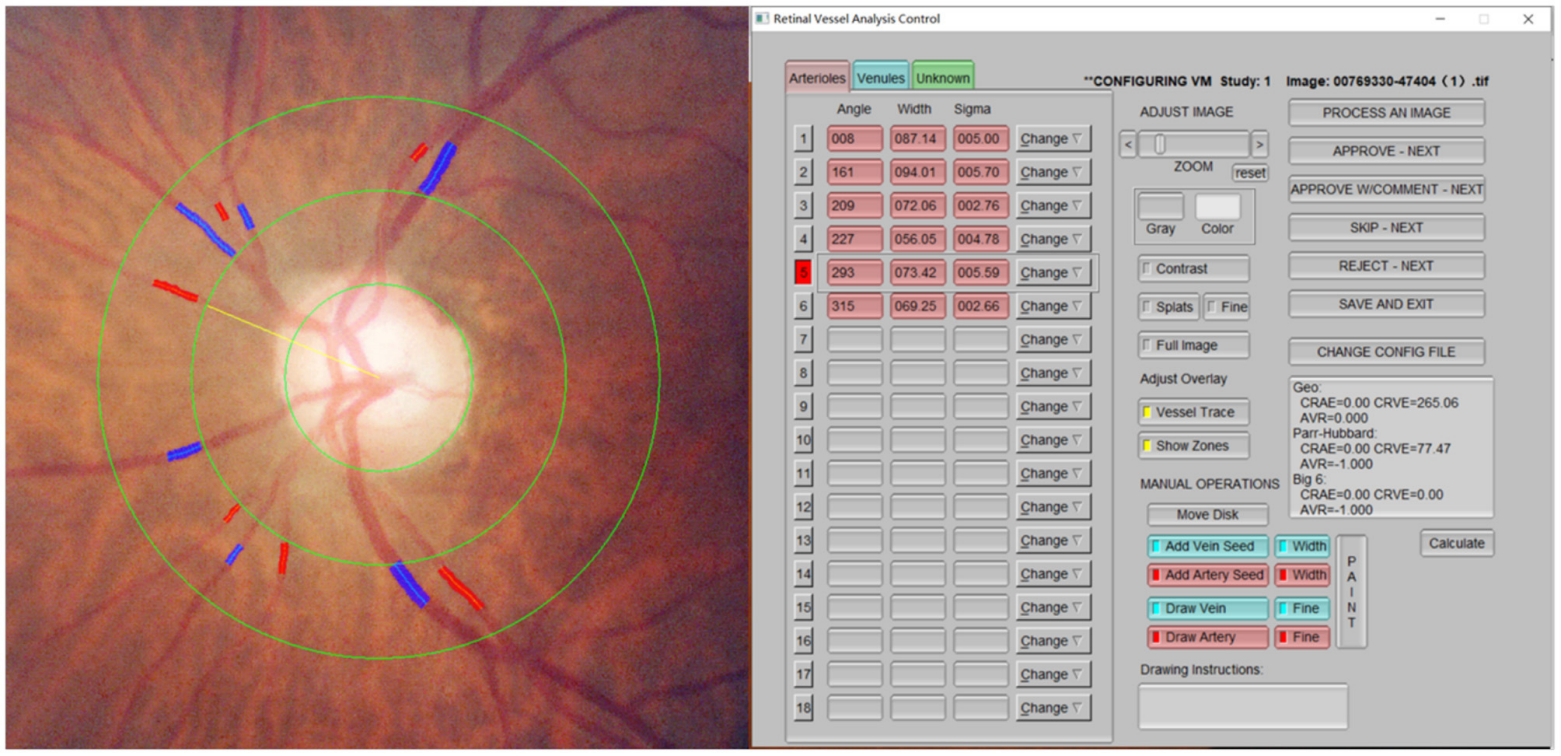
Retinal arteriolar and venular diameters. The diameters of six larger branches of all retinal arteries and veins within the range of 1/2–1 DD from the optic disk were mainly measured.

All subjects were treated with a fundus camera (Kowa, Tokyo, Japan) after pupil dilation. Fundus photos centered on the optic disk were then obtained. The retinal vascular caliber was measured using semiautomated vascular measurement software (IVAN, Department of Ophthalmology Visual Science, University of Wisconsin, Madison, Wis., USA). The diameters of six larger branches of all retinal arteries and veins within the range of 1/2–1 DD from the optic disk were mainly measured (Figure 3). The following parameters were obtained using the calculation formula for the relationship between the retinal vascular branch and main diameter proposed by Knudtson et al. (18) (modified Parr–Hubbard formula): central retinal artery equivalent (CRAE), central retinal vein equivalent (CRVE), and arterio-venous ratio (AVR). Two trained graders completed the measurement in a double-blind manner. Inter-rater agreement was measurred between two graders by intraclass correlation coefficient. The intraclass correlation coefficient for CRAE was 0.884 (95% CI: 0.856–0.907) and 0.905 (95% CI: 0.882–0.923) for CRVE.

### Statistical Analysis

The Statistical Package for Social Sciences, version 26.0, statistical software was used for data statistical analysis. Measurement data were expressed as mean ± standard deviation ($\bar{x}$ ± s). The independent sample *t*-test was used if the data met the normal distribution. Otherwise, the rank-sum test was used. Moreover, the chi-square test was used for counting. Binary logistic regression (forward: conditional) was used to establish the different risk assessment models for stroke. The receiver operating characteristic (ROC) curve of the model was drawn to calculate its sensitivity, specificity, and area under the curve of the model. The Delong test compared area under the curve. A *P* < 0.05 was considered statistically significant.

## Results

### Baseline Data of the Two Groups of Patients

**Figure 4:**
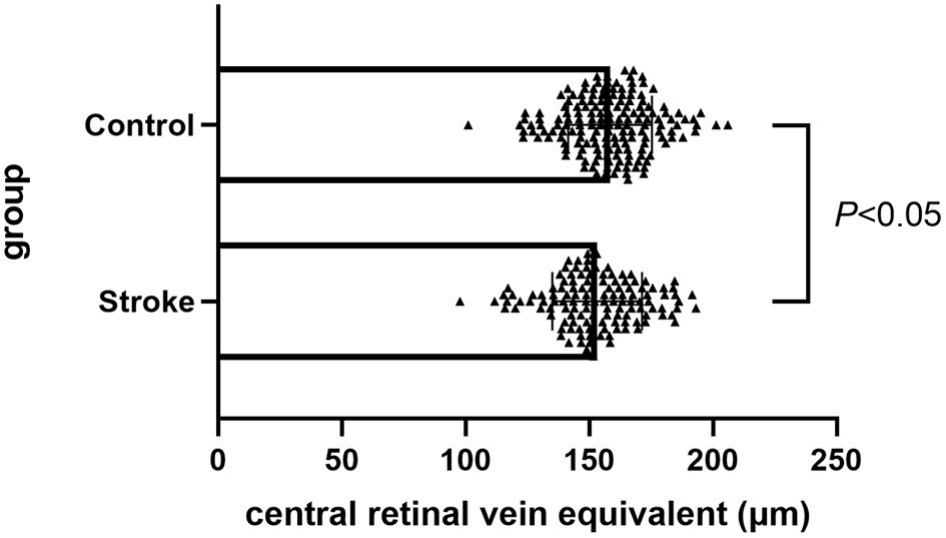
Comparison of CRAE between ischemic stroke group and control group. CRAE, central retinal artery equivalent.

This study included 296 patients (296 eyes), including 128 cases (128 eyes) of ischemic stroke and 168 cases (168 eyes) in the normal control group. No significant differences in age, body mass index, hypertension history, hyperlipidemia, and coronary heart disease history exist between the two groups. The differences in sex, diabetes history, and smoking and drinking history were statistically significant. The CRAE in the ischemic stroke group was lower compared with the normal control group, and the difference was statistically significant (*p* = 0.011) (Figure 4). Moreover, no significant difference was noted between the CRVE and AVR groups (Table 1).

**Table 1:** Baseline characteristics.

	**Ischemic stroke**	**Control**	***p*-value**
Number of eyes (*n*)	128	168	
Age (years)	61.00 ± 8.30	60.42 ± 9.32	0.577
BMI (kg/m^2^)	25.22 ± 3.31	25.39 ± 3.46	0.668
SEX (Male/Female)	98/30	97/71	0.001
Hypertension (%)	87 (68.0%)	101 (60.1%)	0.165
Hyperlipidemia (%)	77 (60.2%)	95 (56.5%)	0.533
Diabetes (%)	56 (43.8%)	38 (22.6%)	0.000
Coronary heart disease (%)	12 (9.4%)	26 (14.3%)	0.120
Smoking (%)	67 (52.3%)	52 (31.0%)	0.000
Drinking (%)	58 (45.3%)	50 (29.8%)	0.006
**WML (%)**			0.009
Grade 0	8 (6.3%)	28 (16.7%)	
Grade I	57 (44.5%)	84 (50.0%)	
Grade II	52 (40.6%)	46 (27.4%)	
Grade III	11 (8.6%)	10 (5.9%)	
CRAE (μm)	153.14 ± 18.12	158.37 ± 16.96	0.011
CRVE (μm)	237.75 ± 29.68	239.56 ± 27.21	0.592
AVR	0.65 ± 0.08	0.67 ± 0.07	0.069

*WML, white matter lesions; CRAE, central retinal artery equivalent; CRVE, central retinal vein equivalent; AVR, arterio-venous ratio*.

### Logistic Regression Model and ROC Curve Analysis

Diabetes [odds ratio (OR), 2.453; 95% confidence interval (CI), 1.468 and 4.098] and smoking (OR, 2.260; 95% CI, 1.388 and 3.681) were closely associated with ischemic stroke in model 1 (the traditional risk factor model of ischemic stroke). Moreover, the CRAE model (OR, 0.983; 95% CI, 0.970 and 0.996) was closely associated with ischemic stroke in model 2. The WML model (OR, 1.623; 95% CI, 1.195 and 2.205) was closely associated with ischemic stroke in model 3. Furthermore, the combined the traditional risk factor, WML and CRAE models (OR, 0.982; 95% CI, 0.968 and 0.996), WML (OR, 1.570; 95% CI, 1.133 and 2.176), diabetes (OR, 2.391; 95% CI, 1.408 and 4.059), and smoking (OR, 2.202; 95% CI, 1.335 and 3.632) were closely associated with ischemic stroke in model 4 (the traditional risk factors; Table 2).

**Table 2:** Different ischemic stroke risk assessment models.

	**B**	**S.E**.	**OR (95% CI.)**	***P*-value**
**Model 1**
Diabetes	0.897	0.262	2.453 (1.468, 4.098)	0.001
Smoking	0.815	0.249	2.260 (1.388, 3.681)	0.001
**Model 2**
CRAE	−0.017	0.007	0.983 (0.970, 0.996)	0.012
**Model 3**
WML	0.485	0.156	1.623 (1.195, 2.205)	0.002
**Model 4**
CRAE	−0.018	0.007	0.982 (0.968, 0.996)	0.013
WML	0.451	0.167	1.570 (1.133, 2.176)	0.007
Diabetes	0.872	0.270	2.391 (1.408, 4.059)	0.001
Smoking	0.789	0.255	2.202 (1.335, 3.632)	0.002

*Model 1: logistic regression of ischemic stroke with traditional risk factors; Model 2: logistic regression of ischemic stroke with CRAE; Model 3: logistic regression of ischemic stroke with WML. Model 4: logistic regression of ischemic stroke with traditional risk factors, WML and CRAE; CRAE, retinal artery equivalent; WML, white matter lesions*.

**Figure 5:**
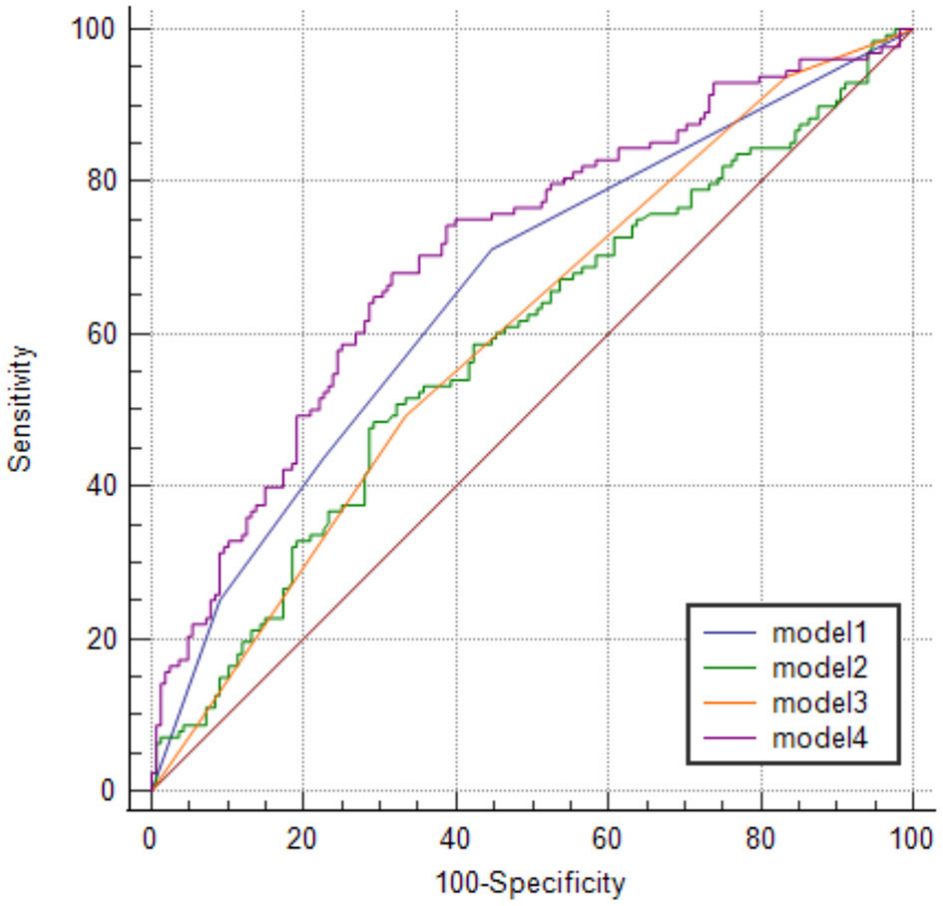
ROC curve analysis of four different risk assessment models.

The sensitivity and specificity of models 1 and 2 were 71 and 55% and 48 and 71% with the area under the curve of 0.658 (95% CI, 0.595–0.721; *p* < 0.0001) and 0.586 (95% CI, 0.520–0.652; *p* = 0.0107), respectively. In model 3, when WML's Fazekas grade ≥ II was used as the threshold for predicting the stroke occurrence stroke, the sensitivity and specificity were 49% and 67%, respectively, with the area under the curve of 0.601 (95% CI, 0.536–0.665, *p* = 0.0009). Furthermore, the sensitivity and specificity of model 4 were 68 and 68.5%, respectively, with an area under the curve of 0.708 (95% CI, 0.648–0.768; *p* < 0.0001; Table 3; Figure 5). The area under the ROC curve in models 1, 2, 3, and 4 showed statistically significant differences. No statistical significance was noted in the pairwise comparison of other models (Table 4).

**Table 3:** ROC curve analysis of four different risk assessment models.

	**AUC**	**95% CI**.	***P*-value**
Model 1	0.658	0.595–0.721	<0.0001
Model 2	0.586	0.520–0.652	0.0107
Model 3	0.601	0.536–0.665	0.0009
Model 4	0.708	0.648–0.768	<0.0001

*Model 1: logistic regression of ischemic stroke with traditional risk factors; Model 2: logistic regression of ischemic stroke with CRAE; Model 3: logistic regression of ischemic stroke with WML. Model 4: logistic regression of ischemic stroke with traditional risk factors, WML and CRAE. CRAE, retinal artery equivalent; WML, white matter lesions*.

**Table 4:** Comparison of different risk assessment models for ischemic stroke.

	**Difference between areas**	**Z statistic**	***P*-value**
Model 1 vs. Model 2	0.0722	1.581	0.1139
Model 1 vs. Model 3	0.0577	1.410	0.1586
Model 1 vs. Model 4	0.0500	2.497	0.0125
Model 2 vs. Model 3	0.0145	0.315	0.7528
Model 2 vs. Model 4	0.1220	3.361	0.0008
Model 3 vs. Model 4	0.1080	3.385	0.0007

*Model 1: logistic regression of ischemic stroke with traditional risk factors; Model 2: logistic regression of ischemic stroke with CRAE; Model 3: logistic regression of ischemic stroke with WML. Model 4: logistic regression of ischemic stroke with traditional risk factors, WML and CRAE. CRAE, retinal artery equivalent; WML, white matter lesions*.

## Discussion

Ischemic stroke is the most common stroke in clinical practice, leading to risk factors, divided into modifiable and non-modifiable (19), which includes sex, age, race, and genetics, which are unpreventable risk factors that cannot be changed in the later stage. Furthermore, high blood pressure, diabetes, dyslipidemia, cardiovascular system diseases, and bad living habits (smoking, drinking alcohol), which are preventable risk factors that can be intervened, are also included. These risk factors are closely related to stroke occurrence or are independent risk factors for stroke (14, 19). Previous reviews have reported that the incidence rate of age-adjusted stroke incidence is lower in women than in men (20). The incidence rate of stroke is higher in China than in women, according to Chinese studies (21, 22). A similar study in Southeast Asia is similar to that in men, and the incidence rate and mortality rate of female stroke are lower (23). In addition, the incidence of stroke is age-specific. The study found that under the age of 75, female stroke is lower than male. Seventy-five or over, twice as many women as men. But after adjusting for age, the risk of stroke was lower in women than in men (24). In this study, the number of male patients with ischemic stroke is more than that of female patients, which reflects the incidence of stroke. Univariate analysis showed that there was significant gender difference between the two groups, and multiple factors were excluded. It may be that the factors of gender and smoking are overlapped (the majority of smokers in China are men). The multivariate analysis finally included smoking and excluded sex factors. This study established the risk assessment model for ischemic stroke based on traditional risk factors, which had low accuracy in predicting stroke risk (area under the curve, 0.658). The sensitivity and specificity were 71 and 55%, respectively.

Moreover, retinal and intracranial vessels share a common embryological, anatomical, and physiological basis (25). The pathological changes of the retinal vessels reflect the changes of small blood vessels in the skull to some extent (26). Therefore, the changes of ocular fundus vessels can be used as an important indicator for the early diagnosis and prediction of cerebrovascular disease risk. A large sample study based on demography has shown that retinal microvascular abnormalities are significantly correlated with the incidence of stroke, which is an important factor in predicting stroke incidence (27, 28). With the development of color photography equipment and the application of computer-aided analysis technology, retinal vascular parameters can be quantitatively analyzed. Quantitative observation of retinal vessels has been used in many population-based stroke studies, but the results of these studies are lack of general consistency. The Atherosclerosis Risk in Communities Study based on demography found that the incidence of stroke increased with the decrease of focal arteriolar narrowing and AVR. People with hypertension were more likely to have this situation than people without hypertension (27). The Cardiovascular Health Study and the Rotterdam Study found that the diameter of retinal vein rather than artery was closely related to stroke (29, 30). Meta analysis also showed that a wider retinal vein diameter could predict stroke (31). In this study, the results showed that the diameter of retinal artery was negatively correlated with the occurrence of stroke, while the diameter of retinal vein and AVR had no significant difference between the two groups. The possible reasons are as follows: firstly, the etiology and classification of stroke are complex. Retinal vascular changes vary according to different stroke subtypes. Different retinal diameter indicators reflect specific cerebral microvascular diseases, such as lacunar stroke with venous dilatation, and other stroke types with arteriolar stenosis (32). Secondly, AVR is the ratio of the diameter of retinal artery and vein, so the result depends on the two factors of artery and vein. The ratio of artery and/or vein is not necessarily different. The control group in this study is not completely healthy patients, but patients with some neurological symptoms excluding acute stroke. Although there is a statistical difference in retinal artery caliber between the two groups, the average difference of crae is only 5 μm. The difference of ARV between the two groups was not statistically significant. While using a single index of retinal artery caliber to establish a risk assessment model for ischemic stroke, the efficiency was low (the area under the curve was 0.586), and the sensitivity and specificity were 49 and 67%, respectively.

WML was first proposed by Canadian neurologists Hachinski et al. (33) in 1986, which referred to the high signal area around the ventricle and subcortex (semi-oval center) on T2-weighted MRI, where the lesion presented diffuse spots or patches with ambiguous edges. According to the lesion site, it could be divided into periventricular WML and deep-brain white matter lesions. Current evidence suggests that WML is more common and severe in patients with ischemic stroke compared with healthy people and is closely associated with stroke and can predict the increased risk of stroke (34, 35). A population-based Rotterdam Study confirmed that changes in white matter microstructure were associated with stroke risk and could improve risk prediction models (36). Furthermore, the Framingham Heart Study was a community-based prospective cohort study that showed an association between severe WML and stroke events. However, continuous quantitative measurement of lesions was not associated with an increased risk of stroke, suggesting that WML was only associated with an increased risk of stroke when it exceeded a certain threshold (37). In this study, the control group was not completely healthy people. Probably due to this reasons, a certain number of patients with higher white matter score appeared in the control group. The results of the current study also suggested that WML was an independent risk factor for stroke. In addition, the WML Fazekas grade in the stroke group was generally higher compared with the non-stroke group, suggesting the more severe WML lesion, i.e., the higher the Fazekas grade, the greater the risk of stroke. When the Fazekas classification of WML was ≥grade II (Fazekas score for 3–4), it was regarded as the critical value for stroke risk with a risk assessment model of the area under the ROC curve of 0.601 and a sensitivity and specificity of 49 and 67%, respectively.

Color fundus imaging is an economical, convenient, and non-invasive examination technique, which is widely used in ophthalmology. Although CT, MRI, and other traditional neuroimaging techniques cannot be replaced, color fundus imaging provides unique information about brain lesions and can be used as a complementary method for stroke assessment. Although previous studies have found that retinal vascular abnormalities are closely related to the occurrence of stroke, retinal examination has the potential to screen and predict stroke, but it has not been used in clinical practice. Patients with stroke often have brain tissue changes before the onset or even a few years ago. Therefore, for high-risk patients with traditional risk factors and white matter lesions in clinic, will retinal screening improve the predictability of stroke? This study found that the traditional risk factors combined with Fazekas score and central retinal artery diameter risk assessment model were better than the single factor model. This method had better accuracy (sensitivity and specificity were 68 and 68.5%, respectively, and the area under the curve was 0.708). This result suggests that the method can better assess the risk of ischemic stroke, contribute to the risk stratification of patients with high risk of stroke. It may improve the early warning and prevention of stroke and optimize patients management.

In addition, there are limitations in this study. The included cases were collected from a single center. The cross-sectional study with a small sample limits our ability to the prediction of stroke. The stroke group was derived from hospitalized patients, and two subtypes of ischemic stroke was studied, and other stroke types were not included. The sample size still needs to be further expanded.

## Data Availability Statement

The raw data supporting the conclusions of this article will be made available by the authors, without undue reservation.

## Ethics Statement

The studies involving human participants were reviewed and approved by the Ethics Committee of Beijing Friendship Hospital, Capital Medical University. The patients/participants provided their written informed consent to participate in this study. Written informed consent was obtained from the individual(s) for the publication of any potentially identifiable images or data included in this article.

## Author Contributions

LZ jointly conceived of the study with BJ, contributed to the statistical analysis, and interpretation of data as well as drafting the manuscript and revising it critically. YW and HH revised the manuscript and provided final version to be published. HL and XY is responsible for the acquisition of data. All authors have given final approval of the version to be published.

## Conflict of Interest

The authors declare that the research was conducted in the absence of any commercial or financial relationships that could be construed as a potential conflict of interest.

## Publisher's Note

All claims expressed in this article are solely those of the authors and do not necessarily represent those of their affiliated organizations, or those of the publisher, the editors and the reviewers. Any product that may be evaluated in this article, or claim that may be made by its manufacturer, is not guaranteed or endorsed by the publisher.
